# Predicting disease associations via biological network analysis

**DOI:** 10.1186/1471-2105-15-304

**Published:** 2014-09-17

**Authors:** Kai Sun, Joana P Gonçalves, Chris Larminie, Nataša Pržulj

**Affiliations:** Department of Computing, Imperial College London, London, SW7 2AZ UK; Computational Biology, GlaxoSmithKline, Stevenage, Hertfordshire, SG1 2NY UK

**Keywords:** Disease classification, Network analysis, Graph theory, Topology, Protein-protein interaction

## Abstract

**Background:**

Understanding the relationship between diseases based on the underlying biological mechanisms is one of the greatest challenges in modern biology and medicine. Exploring disease-disease associations by using system-level biological data is expected to improve our current knowledge of disease relationships, which may lead to further improvements in disease diagnosis, prognosis and treatment.

**Results:**

We took advantage of diverse biological data including disease-gene associations and a large-scale molecular network to gain novel insights into disease relationships. We analysed and compared four publicly available disease-gene association datasets, then applied three disease similarity measures, namely annotation-based measure, function-based measure and topology-based measure, to estimate the similarity scores between diseases. We systematically evaluated disease associations obtained by these measures against a statistical measure of comorbidity which was derived from a large number of medical patient records. Our results show that the correlation between our similarity measures and comorbidity scores is substantially higher than expected at random, confirming that our similarity measures are able to recover comorbidity associations. We also demonstrated that our predicted disease associations correlated with disease associations generated from genome-wide association studies significantly higher than expected at random. Furthermore, we evaluated our predicted disease associations via mining the literature on PubMed, and presented case studies to demonstrate how these novel disease associations can be used to enhance our current knowledge of disease relationships.

**Conclusions:**

We present three similarity measures for predicting disease associations. The strong correlation between our predictions and known disease associations demonstrates the ability of our measures to provide novel insights into disease relationships.

**Electronic supplementary material:**

The online version of this article (doi:10.1186/1471-2105-15-304) contains supplementary material, which is available to authorized users.

## Background

Correct diagnosis is critical for effective treatment and prevention of disease. As a result, disease classification has become a key cornerstone of modern medicine. Disease may be classified by any one of a number of criteria: topographic, anatomic, pathological, physiological, etiological, juristic, epidemiological or statistical approaches. However, without considering the molecular mechanisms driving diseases, such knowledge is limited and can even be misleading. For example, a common phenotype can be caused by different underlying mechanisms, such as breast cancer, which can be divided into several subgroups that are characterized by distinct patterns of pathway activation [1]. However, a common mechanism may lead to different phenotypes. For example, a mutation at the *β*-globin locus may lead to sickle-cell anemia with different phenotypes such as bony infarcts, acute chest syndrome and stroke [2].

During the past decade, a wealth of biological data has been generated from various large-scale genomic studies, prompting the scientific community to gain deeper insight into disease relationships based on their underlying biological mechanisms. Various types of biological data have been used to infer associations between diseases. One of the most commonly used biological data is disease-gene association. In a broad definition, a disease-gene association is a connection reported in the literature, which can be a genetic association (i.e., mutations in that gene may lead to that disease), or a connection inferred from other aspects. Disease-gene associations can be obtained from large-scale knowledge-bases such as the Online Mendelian Inheritance in Man (OMIM) [3]. Early studies used text mining to infer similarities between phenotypes contained in OMIM, and found those similarities were positively correlated with a number of measures of gene functions [4] and could be used to predict disease-causing genes [5]. Also by using OMIM, Goh *et al.* [6] constructed the human diseasome by connecting diseases that share a disease-causing gene. Other types of biological data such as biological pathways [7], gene expression data [8, 9], biomedical ontologies [10, 11], and genome-wide association study (GWAS) data [12–14], have also been used to improve the current understanding of disease relationships from different aspects. Recently, networks have been used to model large-scale biological data, and network topology is beginning to provide insights into diseases and their associations [6, 15–17]. By considering the interconnectivity of biomolecules in the cell, the topology of biological networks is expected to have various biological and clinical applications [18, 19].

Despite these advances, early studies have several limitations when inferring disease associations from biological data. First, some studies only considered several specific diseases, rather than giving a global comparison among all diseases (e.g., [9, 12–14]). This is the case for GWAS-based studies, since a small number of GWAS studies have been completed to date in a relatively small proportion of the total disease population. Furthermore, most studies solely used OMIM as the source of disease-gene association data. OMIM is a catalogue of mendelian disorders and as a result, most diseases are annotated with few genes in OMIM [20]. Limitations of using OMIM have also been discussed previously [21, 22]. Finally, most computationally predicted disease associations were not systematically evaluated due to the difficulty in identifying a suitable benchmark of known disease associations. In particular, most studies were only able to validate part of their results by comparing them with phenotypic similarities (e.g., [12]) or mining the literature manually (e.g., [13]). A comparison of previous studies can be found in Table 1.

**Table 1:** **Comparison of studies on inferring disease-disease associations**

	Data	Size	Evaluation
van Driel *et al.* (2006) [4]	OMIM	5132 phenotypes in OMIM	Comparing results with genotypic similarities
Lage *et al.* (2007) [5]	OMIM	7000 OMIM record pairs	Evaluating results against the overlap of the OMIM record pairs
Goh *et al.* (2007) [6]	OMIM	1284 OMIM diseases	Analysing network topologicalproperties
Huang *et al.* (2009) [12]	GWAS	7 diseases	Comparing results with phenotypic similarities
Li and Agarwal (2009) [7]	Pubmed abstracts,biological pathways	1028 diseases in MeSH	Comparing results with MeSHclassification
Kim *et al.* (2009) [13]	GWAS	53 clinical traits related tosevere asthma	Mining the literature manually
Hu and Agarwal (2009) [8]	Expression data	645 diseases in MeSH	Comparing results with MeSHclassification
Suthram *et al.* (2010) [9]	Expression data, PPI	54 diseases	Evaluating results against genetic similarities
Lewis *et al.* (2011) [14]	GWAS	61 diseases	Comparing results with Huang *et al.*(2009) results
Mathur and Dinakarpandian *et al.*(2007) [10]	DO annotation, GOannotation	36 diseases (for evaluation)	Evaluating results using 68 curated disease associations
Our study	Disease-gene associations, GOannotation, PPI	543 ICD-9 diseases	Evaluating results against ICD-9classification, comorbidity, andgenetic similarities derived fromGWAS data

**Figure 1:**
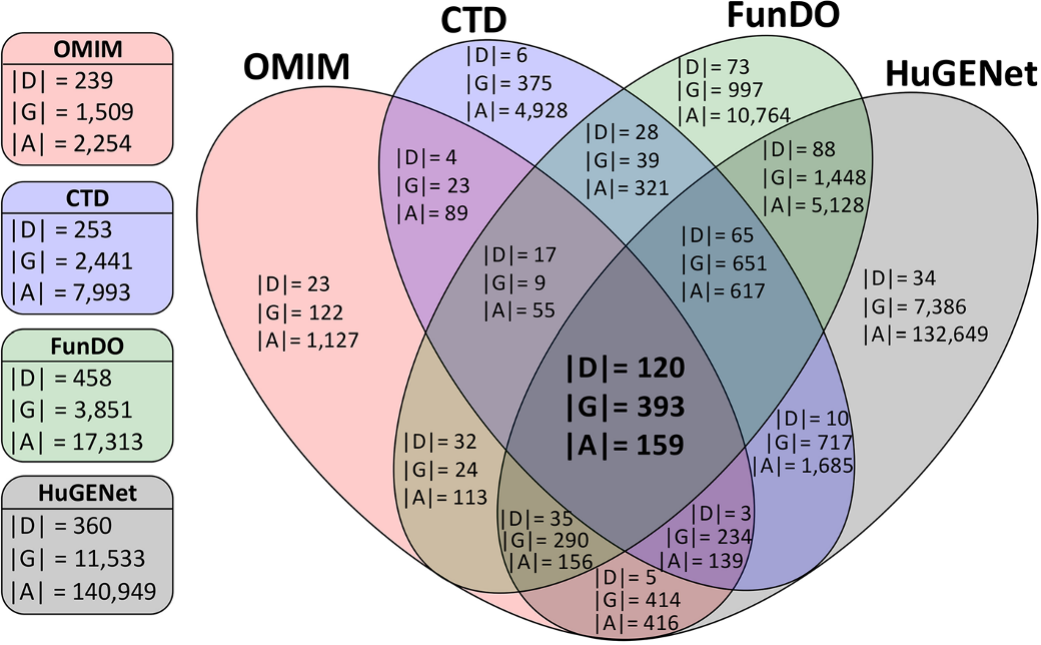
**The overlap of datasets.** The overlap of diseases (denoted by ‘D’), genes (denoted by ‘G’) and their associations (denoted by ‘A’) between the four disease-gene association datasets we analysed. Boxes on the left list the sizes of the datasets. The size of the intersection of the datasets is marked in bold.

The comparison is based on the data used to derive associations (denoted by ‘Data’), number of diseases evaluated (denoted by ‘Size’) and benchmarks used for evaluation (denoted by ‘Evaluation’). The number of diseases evaluated in our study is computed as the union of diseases annotated in the four disease-gene association datasets we analysed, given in Figure 1.

In our study, we used diverse biological data from a number of repositories to gain novel insights into the relationship of over 500 known human diseases by considering their underlying biological mechanisms. We used disease-gene associations obtained from four different sources to avoid the bias introduced by a single dataset. Moreover, we took advantage of the topology of a large-scale molecular network to examine its use for inferring disease associations. We applied three different disease similarity measures, namely annotation-based measure, function-based measure and topology-based measure, to estimate similarity scores between diseases. The disease associations obtained by the three measures were systematically evaluated against the standard disease classification system, namely the International Classification of Diseases, 9^th^ revision (ICD-9) [23], and a statistical measure of comorbidity derived from a large number of medical patient records. In addition, we evaluated our predicted disease associations by using disease associations generated from GWAS studies, which represent one of the most robust routes for identifying causal relationships between genes and diseases. To our knowledge, this is the first time comorbidity and GWAS data have been used to evaluate computationally predicted disease associations.

In the rest of this paper, we will start with a description of the biological data we analysed, followed by details of our methodology of measuring disease associations. Then we will show and discuss the evaluation of disease associations predicted by our similarity measures against known disease associations derived from ICD-9, comorbidity data and GWAS data. Finally, we will present case studies to demonstrate the ability of our similarity measures to predict novel disease associations.

## Methods

### Biological data

Three types of biological data were used in this study: protein-protein interactions (PPIs), Gene Ontology (GO) annotations and disease-gene associations.

#### PPI network

We modelled PPI data as a network. A *network* or *graphG*(*V*,*E*) consists of two types of elements, a set *V* of nodes and a set *E*⊆*V*×*V* of edges connecting them. A PPI network models the physical interaction among proteins in the cell, in which a node represents a protein, and an undirected edge exists between a pair of nodes if their corresponding proteins can physically bind to each other. Currently available PPIs are mostly yielded from various high throughput proteomics experiments, such as yeast two-hybrid screening (e.g., [24]) and affinity capture mass spectrometry (e.g., [25]). We constructed a human PPI network using data obtained from BioGRID [26] version 3.1.93 (released in October 2012). All self-loops, duplicate interactions were removed since we considered only simple, undirected graphs. We also removed the cross-species interactions (i.e., interactions between human proteins and proteins of other species) because we focused on the physical interactions between human proteins in our study. The PPI network we constructed contained 11,375 nodes and 66,317 edges, while its largest connected component contained 11,261 nodes and 66,253 edges. Note that the second largest connected component only contained 5 nodes and 5 edges. There were also 7 isolated triangles and 43 isolated edges in the PPI network. The presentence of these small components may be due to the incompleteness of the PPI data. In addition, the topology of these small components is not as informative as that of the largest connected component. For these reasons, we only used the largest connected component of the PPI network in our analysis.

#### GO annotations

Genes are annotated with GO terms to represent their biological properties [27]. All GO terms are organised in three domains: cellular component, molecular function and biological process. We downloaded the ontology file and annotations of *Homo sapiens* from the Gene Ontology database (http://www.geneontology.org) in November 2012. We removed annotations with evidence code ‘Inferred from Electronic Annotation’ (IEAs), since IEAs are computationally inferred annotations which have not been reviewed by curators. In total, we collected 171,888 annotations between 13,166 genes and 10,787 GO terms.

#### Disease-gene associations

Disease-gene associations can be modelled as a graph containing both known human diseases and disease-related genes in the human genome. The degree of a disease is the number of genes associated with that disease, while the degree of a gene is the number of diseases annotated with that gene. We used four disease-gene association datasets obtained from different sources: OMIM, Comparative Toxicogenomics Database (CTD) [28], Functional Disease Ontology annotations (FunDO) [29] and Human Genome Epidemiology Network (HuGENet) [30]. Among these datasets, OMIM, CTD, and FunDO contain curated associations, while HuGENet contains computationally inferred associations. Details of these disease-gene association datasets are described below.

- OMIM is considered to be the best-curated resource of known phenotype-genotype relationships, and it has been used in various disease-related studies (discussed in the Background section). We downloaded the OMIM database in November 2012. In total, it contains 3,537 diseases (annotated by OMIM IDs), 2,862 genes and 4,337 disease-gene associations.
- CTD provides scientific data describing relationships between chemicals, genes, and human diseases, with the goal of improving the understanding of environmental chemicals’ effects on human health. It contains both curated and inferred disease-gene associations, but we only used curated associations as they have higher confidence than inferred associations. Disease-gene associations directly derived from OMIM were excluded to reduce the dependency between datasets. We downloaded the data from CTD in November 2012 and obtained 17,754 associations between 2,761 diseases (annotated by Medical Subject Heading (MeSH) terms [31]) and 5,828 genes.
- FunDO contains disease-gene associations extracted from the NCBI Gene Reference Into Function (GeneRIF) database. A GeneRIF is a brief statement about the function of a gene, along with information of its association with diseases. We downloaded the latest stable version of FunDO (released in October 2008) and obtained 1,854 diseases (annotated by Disease Ontology (DO) terms), 4,781 genes and 28,442 disease-gene associations.
- HuGENet is known as an integrated knowledge-base on human genome epidemiology. The Phenopedia collection [30] of HuGENet contains disease-gene associations obtained by text-mining of abstracts on PubMed using machine learning techniques. Disease-gene association data were downloaded via HuGE Navigator in September 2012. We obtained 353,883 associations between 2,387 diseases (annotated by Unified Medical Language System (UMLS) [32] Concept Unique Identifiers (CUIs)) and 11,915 genes.

Since disease names or IDs used in these datasets are based on different labelling schemes, we mapped all disease names or IDs to ICD-9 codes, for the purpose of comparing these datasets and further evaluation (also see the Results and discussion section for details). We used the mapping manually constructed by [6] and [33] to convert OMIM IDs to ICD-9 codes, and used the corresponding mapping provided in Disease Ontology version 3 (the latest stable version of DO, released in May 2007) to map DO IDs, MeSH terms and UMLS CUIs to ICD-9 codes. In total, 1,467 OMIM IDs in OMIM, 423 MeSH terms in CTD, 806 DO IDs in FunDO and 693 UMLS CUIs in HuGENet were mapped to ICD-9 codes.

### Disease similarity measures

We applied three similarity measures to estimate similarity scores between diseases. These measures include standard methods (i.e., Jaccard index) and novel measures proposed in this study (i.e., graphlet-based measure). Considering the information used in calculation, the similarity score of a pair of diseases was measured in three different ways: annotation-based, function-based and topology-based.

#### Annotation-based measure

The annotation-based measure solely used the information obtained from disease-gene association data. We applied the Jaccard index, which is known as a standard method for comparing the similarity between two sets, to estimate the similarity score between diseases as follows. Let  be the set of genes associated with a disease *D*_*i*_. We computed the annotation-based similarity score of two diseases *D*_*i*_ and *D*_*j*_ as the Jaccard index (or Jaccard similarity coefficient) of  and :
1

#### Function-based measure

The function-based similarity measure used both GO term annotations and disease-gene associations to estimate the similarity score between a pair of diseases. We first propagated the GO annotations upwards through the GO hierarchy, i.e., when a gene was annotated with a GO term, we assumed associations between the gene and the term’s parents. For each disease *D*_*i*_ annotated in a specific disease-gene association dataset, we then identified the set of GO terms that were overrepresented within , denoted by . The statistical significance (*p*-value) of the enrichment of a GO term was computed according to the hypergeometric distribution for sampling without replacement, and was corrected for multiple testing using the Benjamini-Hochberg test. Only overrepresented GO terms from the ‘biological process’ domain of GO and having a *p*-value less than 0.05 were considered to be in . For a pair of diseases *D*_*i*_ and *D*_*j*_, we computed the Jaccard index of  and  as their function-based similarity score, defined as:
2

#### Topology-based measure

Many studies have shown the relationship between topological properties of proteins in the PPI network and the involvement of proteins in diseases [6, 34, 35]. Topological similarities of proteins in a PPI network are considered as a complementary information to sequence similarities [36]. Thus in this study, we took advantage of the topology of the human PPI network along with disease-gene association data to examine the use of network topology for uncovering novel disease associations. In particular, we proposed a measure to estimate the similarity score between a pair of diseases based on the topological similarity of their annotated genes.

We applied a graphlet-based method to assess the topological similarity of genes in the human PPI network. A *graphlet* is defined as a small, connected and induced subgraph of a larger network [37]. Within each graphlet, some nodes are topologically identical to each other, and such identical nodes are said to belong to the same *automorphism orbit* [38]. The *graphlet signature* of a node *u* is a 73-dimensional vector, whose *i*^th^ element *u*_*i*_ counts the number of times the node *u* is touched by the particular automorphism orbit *i* [39]. According to [39], the signature similarity of a pair of nodes *u* and *v* is defined as:
3

where *w*_*i*_ is a weight assigned to orbit *i* defined as 1−*log*(*o*_*i*_)/*log*(73) (*o*_*i*_ is the dependency count of orbit *i*, see [39] for details). *SigSim*(*u*,*v*) ranges between 0 and 1, where the value of 1 means that the two nodes, *u* and *v*, are considered to be topologically identical. This measure is a highly constraining measure of local topological similarity between two nodes in a network as it compares the nodes based on local structures of their neighbourhoods, which describe their interconnectivities out to a distance of four [39]. Signature similarities have been applied to measure the topological similarities between proteins in a PPI network [34, 36, 39–43]. It has been shown that topologically similar proteins are likely to belong to the same protein complexes, perform the same biological functions, be localised in the same subcellular compartments and have the same tissue expressions [39]. Signature similarities have also been used to relate the network structure around a protein in a PPI network to homology [36] and its involvement in diseases [34]. For these reasons, we hypothesize that the topology around disease genes in the PPI network can reflect the underlying biological mechanisms of diseases.

We calculated the signature similarity of each pair of genes in the human PPI network. Note that the network has an edge density (the proportion of the number of edges to the maximum possible number of edges) of 0.001, which for its size (11,261 nodes and 66,253 edges) is dense enough to avoid low edge density regions in which the topology of networks is unstable (see [44] for details). Here we extended the use of graphlet-based method to measure disease similarities. We introduced two terms to quantify the topology-based similarity score between diseases *D*_*i*_ and *D*_*j*_. The first term, denoted by *AllSig*, is the maximum of the signature similarity between a gene in  and a gene in :
4

The second term, denoted by *ShareSig*, focuses on the topological similarity between genes shared with both diseases:
5

Finally we defined the topology-based similarity score between *D*_*i*_ and *D*_*j*_ as the average of these two terms:
6

### Evaluation

#### Comorbidity associations of diseases

The availability of electronic patient records facilitates studies into disease comorbidity, which indicates the potential for co-occurrence of two given diseases in the same individual. Comorbidity can be considered as a type of disease association derived from electronic medical record, but the underlying driver for comorbidity may be very different from one another. Comorbidity and its correlation with other types of disease associations such as genetic associations [45] and evolutionary associations [46] have previously been studied. Unlike these studies, we used comorbidity data to evaluate disease associations predicted by our similarity measures. Comorbidity associations were downloaded from the Human Disease Network (HuDiNe, [47]), which were obtained from the disease history of 32 million American patients. Diseases were annotated using ICD-9 codes in HuDiNe, and as many diseases in patient records were not specific enough to map to 4-digit or 5-digit codes, we used the comorbidity data annotated using 3-digit level ICD-9 codes for our analysis. The strength of comorbidity association between a pair of diseases can be measured by the Relative Risk and *ϕ*-correlation [47]. Because comorbidity associations quantified by *ϕ*-correlation were reported to contain more connections across different ICD-9 categories [47], we chose *ϕ*-correlation as the measure of comoridity. The *ϕ*-correlation score between *D*_*i*_ and *D*_*j*_ was defined as the Pearson’s correlation for binary variables, given by:
7

where *C*_*ij*_ is the number of individuals affected by both *D*_*i*_ and *D*_*j*_, *N* is the total number of individuals in the population, *P*_*i*_ and *P*_*j*_ are the prevalences of *D*_*i*_ and *D*_*j*_ respectively. A *ϕ*-correlation higher than 0 indicates the co-occurrence of *D*_*i*_ and *D*_*j*_ is more frequently than expected by random. The statistical significance of *ϕ*-correlation was determined by using a *t*-test,
8

where *n*=*max*(*P*_*i*_,*P*_*j*_) is the number of observations used to calculate *ϕ*. We used significant associations at 5% level (*t*≥1.96) for our analyses.

#### GWAS data

GWAS is a powerful method to identify genetic variations associated with diseases and is one of the most robust routes for identifying causal relationships between genes and diseases [48, 49]. GWAS studies examine the genome for single-nucleotide polymorphisms (SNPs) that occur more frequently in people with a particular disease than in people without it. GWAS studies have enabled exploration of gene association in complex diseases in a systematic way on a genome scale. Whilst individual studies are extremely powerful, only a small number of diseases have been studied thus far using GWAS. Hence the GWAS database as a whole is only able to contribute a relatively small component to the overall knowledge base of general disease-gene associations. For this reason, we did not use GWAS data as a source of disease-gene association to measure disease similarity scores, but used them to evaluate our predicted disease associations. We downloaded GWAS data from the National Human Genome Research Institute (NHGRI) GWAS catalog [50] in May 2013. This resource collects significant associations between traits (or diseases) and SNPs from the literature. Similar to [51], we only considered highly confident associations with *p*-value lower than 10^−7^. We also eliminated not replicated associations to minimise false-positives. For all disease-SNP associations in our analysis, we used the corresponding disease-gene associations reported by the authors in the original publications as recorded in the GWAS Catalog. After mapping diseases to ICD-9 codes, we obtained 1,756 genetic associations (from 478 publications) between 126 diseases and 1,298 genes.

## Results and discussion

### Comparison of disease-gene association datasets

We analysed four different disease-gene association datasets: three curated datasets, namely OMIM, CTD and FunDO, and one computationally predicted dataset, HuGENet (details of these datasets can be found in the Methods section). Although these datasets focus on different aspects of the connections between diseases and genes, they are not fully independent since information contained in these datasets is extracted from the literature. For example, disease-gene associations contained in CTD and FunDO were extracted from 9,269 and 48,436 publications respectively, and they have 799 publications in common. We mapped all disease names or IDs annotated in these datasets to ICD-9 codes for a correct comparison (see the Methods section for more details). If several diseases were mapped to a common ICD-9 code, we assigned the union of genes associated with those diseases to that ICD-9 code. In order to evaluate our measures using comorbidity data, we further limited the ICD-9 codes to 3-digit level. We are aware that noise may be introduced when merging diseases into 3-digit level. Generally speaking, a 3-digit level ICD-9 code is always associated with more than one disease, thus the average degree of diseases increased after mapping. Note that it is possible that two diseases may share clinical traits but have different 3-digit level ICD-9 codes, e.g., acute bronchitis (ICD-9: 466) and chronic bronchitis (ICD-9: 491). However, in most cases if two diseases have different ICD-9 codes at 3-digit level, they always have different clinical phenotypes and they are unlikely to share similarity traits.

Interestingly, the overlap among the four disease-gene association datasets is unexpectedly small, as shown in Figure 1. While a considerable number of diseases (120 diseases in total, that is, 50.21%, 47.43%, 26.20% and 33.33% of diseases annotated in OMIM, CTD, FunDO and HuGENet, respectively) have gene annotations in all four datasets, few disease-gene associations (159 associations in total, that is, 7.05%, 1.99%, 0.92% and 0.11% of associations in OMIM, CTD, FunDO and HuGENet, respectively) can be found in all datasets. Additional file 1: Figure S1 further demonstrates the difference between these datasets according to the degree distribution of diseases. In general, these distributions follow power law distributions, indicating that most human diseases are associated with only a few disease genes, while a small number of diseases relate to many genes. However, this scale-free topology may also be an artifact of sampling: several diseases are better studied than others [52]. We notice that in OMIM, most diseases are associated with fewer genes compared with other datasets. The average number of genes associated with a disease in OMIM is 9.43, while in the two other curated datasets CTD and FunDO, these numbers are 31.59 and 37.80. On the other hand, on average a disease in HuGENet is annotated with more than 300 genes: HuGENet has a higher false positive rate compared to other datasets, since its associations were derived from computational predictions rather than manual curations.

The difference and inconsistency discussed above indicate that currently available disease-gene association datasets are still noisy and incomplete. The incompleteness may be due to the focus of the datasets and the nature of the curation process. For example, OMIM mainly focuses on mendelian diseases and traits. Meanwhile, many false positives may be introduced by text-mining the literature (e.g., HuGENet). However, there is no single standard and systematic method to assess the quality of these data. Therefore, to gain a more comprehensive view of human diseases and to test the robustness of our methods, we used all four disease-gene association datasets along with the intersection/union of the three curated datasets in further computation and evaluation.

### Evaluation of similarity measures

#### Correlation with ICD-9

The results obtained by these measures were first evaluated against the standard disease classification system ICD-9. We say that two diseases are associated according to ICD-9, if they are classified under the same ICD-9 category. For example, diabetes mellitus (ICD-9 code: 250) and thyroiditis (ICD-9 code: 245) are classified under the same category ‘endocrine, nutritional and metabolic diseases, and immunity disorders’. To investigate the correlation between our similarity measures and the ICD-9 classification, we tested whether a pair of diseases from the same ICD-9 category tends to have a higher similarity score than diseases from different ICD-9 categories (Table 2). Since similarity scores obtained by our measures are not normally distributed, we used a non-parametric test, namely the Mann-Whitney U test, to assess the statistical significance (*p*-value). Our results show that for all three similarity measures and all four disease-gene association datasets, similarity scores of diseases from the same ICD-9 category are significantly higher than those from different ICD-9 categories.

**Table 2:** **Evaluation of our measures against ICD-9 classification**

Data	Group	Annotation-based	Function-based	Topology-based
OMIM	Same	0.0114 ± 0.0665	0.0355 ± 0.0892	0.4349 ± 0.1101
	Different	0.0010 ± 0.0139	0.0118 ± 0.0314	0.3996 ± 0.0760
	*P*-value	1.2785 ×10^−13^	1.0423 ×10^−52^	2.1257 ×10^−54^
CTD	Same	0.0361 ± 0.1590	0.0728 ± 0.1754	0.4863 ± 0.1770
	Different	0.0050 ± 0.0274	0.0333 ± 0.0662	0.4408 ± 0.1368
	*P*-value	1.4887 ×10^−23^	1.4040 ×10^−9^	2.0240 ×10^−25^
FunDO	Same	0.0418 ± 0.1344	0.0991 ± 0.1611	0.5560 ± 0.2214
	Different	0.0100 ± 0.0262	0.0549 ± 0.0830	0.4952 ± 0.1636
	*P*-value	1.7609 ×10^−144^	9.6708 ×10^−100^	2.7037 ×10^−90^
HuGENet	Same	0.0931 ± 0.1798	0.2470 ± 0.2123	0.8031 ± 0.2248
	Different	0.0438 ± 0.0566	0.1881 ± 0.1522	0.7837 ± 0.2292
	*P*-value	1.4585 ×10^−74^	9.9053 ×10^−72^	4.5910 ×10^−14^
Intersection	Same	0.0338 ± 0.1511	0.0593 ± 0.1907	0.3826 ± 0.1131
	Different	0.0024 ± 0.0329	0.0089 ± 0.0428	0.3496 ± 0.1020
	*P*-value	2.2667 ×10^−2^	2.7448 ×10^−4^	5.4716 ×10^−4^
Union	Same	0.0350 ± 0.1179	0.0963 ± 0.1463	0.5680 ± 0.2226
	Different	0.0085 ± 0.0219	0.0583 ± 0.0818	0.5042 ± 0.1716
	*P*-value	1.3493 ×10^−211^	7.1478 ×10^−113^	4.1709 ×10^−141^

Numbers in the table are similarity scores between diseases from the same ICD-9 categories, compared with those from different ICD-9 categories. *P*-values are calculated by using the Mann −Whitney U test.

#### Correlation with comorbidity

As the goal of our study is to uncover novel disease associations that may reflect common underlying mechanisms, we are more interested in the associations between diseases that belong to different ICD-9 categories. For this reason, we systematically evaluated our similarity measures against a statistical measure of comorbidity. We say two diseases are associated according to comorbidity if they are reported to have a significant co-occurrence in the same individual. In particular, their *ϕ*-correlation score should be higher than a chosen threshold and statistically significant at 5% level. Additional file 1: Figure S2 shows the distribution of *ϕ*-correlation scores for all pairs of diseases we analysed. Note that even though the comorbidity associations we used for evaluation contained disease associations across different ICD-9 categories, there was overlap between associations derived from ICD-9 and comorbidity associations. For example, the association between diabetes mellitus and obesity was supported by both ICD-9 classification and comorbidity data. Since ICD-9 and comorbidity describe the relationship between diseases from different aspects, we believe the evaluations against ICD-9 classification and comorbidity do not contradict each other, but are complementary to each other.

**Figure 2:**
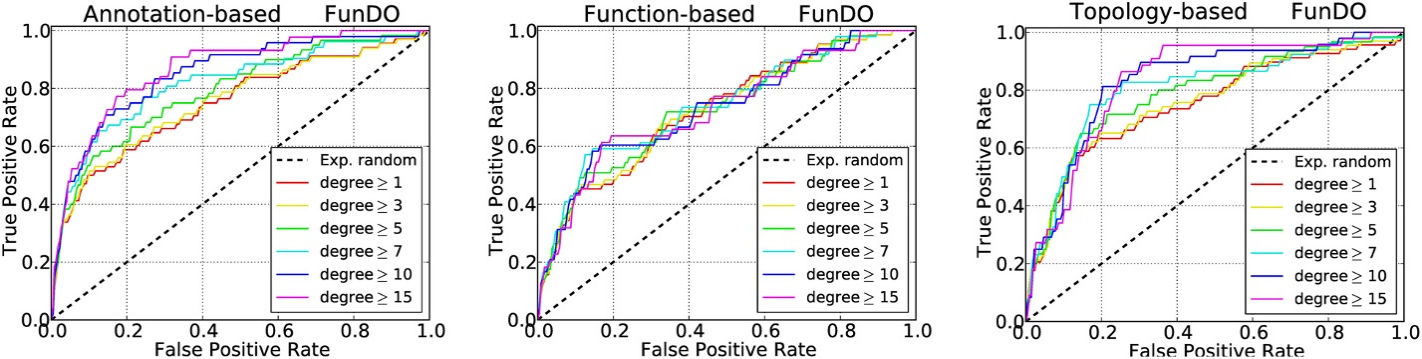
**Evaluation against comorbidity.** ROC curves obtained by evaluating the three disease similarity measures against comorbidity. Due to space limitations, only ROC curves of FunDO are shown here (see Additional file 1: Figure S5 for ROC curves of other datasets). The *ϕ*-correlation threshold was set to 0.06 (the same threshold was used in [47]). We evaluated diseases annotated with at least 1, 3, 5, 7, 10, 15 genes, shown by curves with different colours in each plot.

To assess the ability of our measures to uncover highly confident comorbidity associations, we used Receiver Operating Characteristic (ROC) curves, in which we plotted the *True Positive Rate* (TPR, also known as *sensitivity*) versus the *False Positive Rate* (FPR, also known as 1−*specificity*) for different thresholds of similarity score. TPR is defined as the fraction of true positives (that is, all pairs of diseases having a similarity score higher than a chosen threshold and having comorbidity association) out of the positives (all pairs of diseases having comorbidity association), while FPR is defined as the fraction of false positives (all pairs of diseases having a similarity score higher than a chosen threshold but having no comorbidity association) out of the negatives (all pairs of diseases excluding those having comorbidity association). Figure 2, Additional file 1: Figure S5 and Table 3 show the ROC curves and Area Under Curve (AUC) values obtained by the three disease similarity measures. To illustrate that our results cannot be obtained by chance, we assigned a randomised score which was drawn from the same distribution of the similarity scores to each pair of diseases, and evaluated associations derived from these randomised scores against comorbidity. We show that the correlation between our similarity measures and comorbidity scores is substantially higher than expected at random for all disease-gene association datasets we analysed. In particular, diseases yielding a high similarity score are very likely to have comorbidity associations, thus confirming that our measures are able to uncover known comorbidity relationships.

**Table 3:** **Evaluation of our measures against comorbidity**

Data	Annotation-based	Function-based	Topology-based
OMIM	0.8009 ± 0.0277 (0.5740)	0.8694 ± 0.0073 (0.5120)	0.8495 ± 0.0011 (0.5044)
CTD	0.7849 ± 0.0164 (0.5404)	0.7316 ± 0.0046 (0.5047)	0.7949 ± 0.0042 (0.5203)
FunDO	0.7426 ± 0.0088 (0.4672)	0.7142 ± 0.0017 (0.4940)	0.7497 ± 0.0016 (0.5031)
HuGENet	0.7563 ± 0.0001 (0.5084)	0.8185 ± 0.0001 (0.4987)	0.7153 ± 0.0015 (0.4922)
Intersection	0.9925 ± 0.0001 (0.6013)	0.9802 ± 0.0001 (0.5081)	0.9958 ± 0.0041 (0.4664)
Union	0.8225 ± 0.0045 (0.4704)	0.7491 ± 0.0001 (0.4999)	0.7939 ± 0.0022 (0.5008)
Average	0.8194 ± 0.0837 (0.5270)	0.8106 ± 0.0930 (0.5029)	0.8163 ± 0.0907 (0.4979)

Numbers in the table are AUC values obtained by evaluating the three disease similarity measures against comorbidity associations. The *ϕ*-correlation threshold was set to 0.06 (the same threshold was used in [47]), and all diseases annotated with at least 3 genes were evaluated. Average AUC values obtained by using randomised scores are shown by numbers in brackets (standard deviations are not shown in the table due to space limitation). Each evaluation test was run 30 times to compute the statistics reported in the table.

While varying the *ϕ*-correlation threshold, we obtained higher AUC values for higher thresholds (the ROC curves are not shown in the paper due to space limitations). For example, when the *ϕ*-correlation threshold was set to 0.06 (49 comorbidity pairs), the AUC value was 0.7580 ± 0.0024 (using the topology-based measure and FunDO as the source of disease-gene associations). When the *ϕ*-correlation threshold was set to 0.08 (33 comorbidity pairs) and 0.10 (25 comorbidity pairs), the AUC value increased to 0.7669 ± 0.0027 and 0.7996 ± 0.0060, respectively. This indicates our similarity measures tend to detect strong comorbidity associations with high *ϕ*-correlation. Meanwhile, when we decreased the number of false negatives in the comorbidity data by lowering the *ϕ*-correlation threshold from 0.06 to 0.02, the AUC values we obtained were still higher than expected at random. For example, when the *ϕ*-correlation threshold was set to 0.04 (93 comorbidity pairs) and 0.02 (300 comorbidity pairs), the AUC values we obtained were 0.7064 ± 0.0019 and 0.6017 ± 0.0015, respectively. These results suggest our similarity measures are robust to high false negatives in the comorbidity data. Better ROC curves can also be obtained by evaluating diseases annotated with higher numbers of genes (Additional file 1: Figure S5). From Table 3, we observed that best performances of our similarity measures are achieved by using highly confident curated disease-gene associations (i.e. the intersection set of OMIM, CTD and FunDO), with AUC values higher than 0.98.

Note that our approach is robust to the incompleteness presented in disease-gene association datasets and PPI networks. We downloaded the disease-gene association data from OMIM and the PPI data from BioGRID (version 3.2.112) in June 2014 to re-examine whether we obtained the same results when we used the latest biological data. In total, the OMIM data contained 4,002 diseases (annotated by OMIM IDs), 3,218 genes and 4,816 disease-gene associations. The PPI network we constructed contained 14,089 nodes and 126,891 edges. By re-computing the similarity scores and evaluating the results against comorbidity on these latest biological data, we showed that we were able to obtain results (shown in Additional file 1: Figure S4) that agree with the ones reported in Table 3 and Additional file 1: Figure S5. These results further validated the robustness of our approach.

#### Correlation with GWAS data

We further examined the correlation between our predicted disease associations and currently available highly confident GWAS data (see the Methods section for details) to see whether our findings are supported by GWAS studies. A gene is said to be associated with a disease according to GWAS, if the occurrence of genetic variants (SNPs) within that gene is significantly higher in people with that disease than in people without it. We say that two diseases are associated according to GWAS if they share at least one gene in GWAS data. Since disease-gene associations collected in the four datasets we analysed were extracted from the literature, genetic associations reported in GWAS studies may also be collected in these datasets. To avoid bias in evaluation, we chose FunDO as the source of disease-genes associations, as it has few overlap with GWAS data. In particular, since most GWAS data were published after FunDO’s last stable release (October 2008), only 42 out of 48,436 publications in FunDO were also found in GWAS data. We removed disease-gene associations collected from the common 42 publications before computing similarity scores between diseases using FunDO. Similar to our evaluation against comorbidity, we used ROC curve analysis to assess the ability of our similarity measures to recover disease associations derived from GWAS (Table 4). For each of the three measures, we found that the correlation between our similarity measures and GWAS data is substantially higher than expected at random. This result further confirms the validity of our methods.

**Table 4:** **Evaluation of our measures against GWAS**

Data	Annotation-	Function-	Topology-
	based	based	based
F/G	0.7224 ± 0.0010	0.6781 ± 0.0001	0.6863 ± 0.0009
	(0.4945)	(0.4968)	(0.5005)
Common	0.7527 ± 0.0010	0.7147 ± 0.0001	0.7555 ± 0.0020
	(0.4926)	(0.5005)	(0.4951)

Numbers in the table are AUC values obtained by evaluating the three disease similarity measures against disease associations derived from highly confident GWAS data. Only diseases annotated with at least 3 genes were evaluated. ‘F/G’ are diseases having associated genes in both FunDO and GWAS data (99 diseases in total). ‘Common’ are diseases having associated genes in all four disease-gene association datasets (given in Figure 1) and GWAS data (50 diseases in total). Average AUC values obtained by using randomised scores are shown by numbers in brackets (standard deviations are not shown in the table due to space limitation). Each evaluation test was run 30 times to compute the statistics reported in the table.

### Comparison of similarity measures

The three similarity measures, namely annotation-based measure, function-based measure, and topology-based measure, use different biological information to predict disease associations. For a pair of diseases, the annotation-based measure estimates their similarity score based on the overlap of their annotated genes, while the function-based measure estimates their similarity score based on the overlap of their associated biological functions derived from GO annotations. The topology-based measure makes use of the topology information derived from the underlying PPI network, and estimates disease similarity scores based on the topological similarity of their annotated genes. Based on our evaluation, the three similarity measures perform well in recovering known disease associations. Note that since all three measures compare diseases based on information derived from their associated genes, the three measures are not independent from each other. Diseases that have many shared genes are likely to have common biological processes and have high topological similarities. In addition, a part of the GO annotations is inferred from PPIs (i.e., annotations with evidence code ‘inferred from physical interactions’). However, even though dependency between the three measures exists, the three measures uncover different aspects of disease-disease associations. In fact, the predictions derived from them can differ from each other, demonstrating that the three measures give different insights despite being dependent. Additional file 1: Figure S3 shows the overlap of disease associations predicted by the three measures. When considering the top 5% of the most associated disease pairs as our predicted disease associations, 14% ∼ 38% of the predictions are supported by all three similarity measures.

In the topology-based measure, we used two terms, namely *AllSig*(*D*_*i*_,*D*_*j*_) and *ShareSig*(*D*_*i*_,*D*_*j*_), to measure the topological simialrity of disease genes. Since the term *AllSig*(*D*_*i*_,*D*_*j*_) is defined as the maximum of the signature similarity between a gene associated with disease *D*_*i*_ and a gene associated with disease *D*_*j*_, we have *AllSig*(*D*_*i*_,*D*_*j*_)=1 if the two diseases *D*_*i*_ and *D*_*j*_ have at least one common genes. The term *ShareSig*(*D*_*i*_,*D*_*j*_) is defined as the maximum of the signature similarity between genes that are shared between diseases *D*_*i*_ and *D*_*j*_, thus we have *ShareSig*(*D*_*i*_,*D*_*j*_)=0 if the two diseases share no genes. Therefore, the topology-based similarity score for a pair of diseases that share genes is always higher than a pair of diseases that do not share genes. To assess the contribution of the two terms, *AllSig* and *ShareSig*, in predicting disease associations, we evaluated the performance of the topology-based similarity measure for predicting comorbidity associations by solely using *AllSig* and *ShareSig* as the disease similarity score. The good performance of the topology-based similarity measure is mainly attributed to the term *AllSig* when using OMIM or CTD as the disease-gene association dataset (Additional file 1: Table S3). Since in these two datasets, only 2.69% (OMIM) and 16.62% (CTD) disease pairs have common genes, we have *Sim*_*topology*_=*AllSig* for most disease pairs. On the other hand, the good performance of the topology-based measure is mainly caused by *ShareSig* when using FunDO or HuGENet, as in these two datasets 31.41% (FunDO) and 80.57% (HuGENet) of disease pairs have common genes.

Our similarity measures are sensitive to the noise in disease-gene association data. We notice that prediction performances of our similarity measures generally decrease with the increase of noise level, thus using the intersection of curated disease-gene association datasets results in the best performance when predicting comorbidity associations (Table 3 and Additional file 1: Figure S5). Both the annotation-based measure and the topology-based measure have better performances by using curated disease-gene associations (i.e., OMIM, CTD and FunDO) than computationally predicted associations (i.e., HuGENet). However, the function-based measure obtains lower AUC values for curated datasets CTD and FunDO than the two other similarity measures, but higher AUC values for HuGENet. In this regard, the function-based measure may be more appropriate for analysing predicted datasets, while the annotation-based measure and topology-based measure may be more appropriate for analysing curated datasets.

The annotation-based measure is straightforward, but has relatively good performance according to our evaluation. However, as it only uses disease-gene associations to estimate similarity scores, for a pair of diseases sharing few genes, their annotation-based similarity score may be low, even if their annotated genes are closely related. In particular, the annotation-based measure is highly affected by the occurrence of pleiotropic genes (genes that cause multiple phenotypes) in the dataset. We obtained the list of 802 pleiotropic genes from the OMIM Morbidmap by identifying genes that associated with more than one disease (similar approach was used in [53]). To examine the influence of pleiotropic genes on our measures, we excluded these genes from OMIM and evaluated the performances of our similarity measures against comorbidity. Note that when pleiotropic genes were excluded from OMIM, there were no disease pairs that had any common genes. Therefore, the annotation-based similarity score for a pair of diseases became 0 in this case and no predictions could be derived from the annotation-based measure. On the other hand, since both the function-based measure and the topology-based measure use additional data sources (GO annotations or network topology) to estimate similarity scores, they are less affected by pleiotropic genes. AUC values obtained by the function-based measure and the topology-based measure dropped to 0.7816 and 0.7199 respectively, after removing pleitropic genes from OMIM. These results show the contribution of similarities between specific genes (genes associated with only one disease) to the prediction performances of our similarity measures.

Since disease-gene association datasets were obtained by different research groups and approaches, good performances for all datasets confirm the robustness of our similarity measures in predicting disease associations. In addition, the topology-based measure is also robust to the noise and incompleteness presented in PPI networks. We evaluated this by using PPI data obtained from different releases of BioGRID database (see Additional file 1: Table S1 for details). Generally speaking, the performance of the topology-based measure slightly decreases when using early PPI networks (Additional file 1: Table S2). However, AUC values obtained by using these early PPI networks are still substantially higher than expected at random. These results suggest that the ability of the topology-based measure to predict disease-disease associations may increase with more accurate and complete PPI data.

### Case studies

To demonstrate how our similarity measures can be used for uncovering novel disease associations, we present a case study for diabetes mellitus (DM, ICD-9 code: 250). DM is a metabolic disease that affects the body’s ability to produce or use insulin, a hormone for regulating carbohydrates. It causes hyperglycemia and may lead to severe consequences such as brain damage, amputations and heart disease [54]. Table 5 lists the top 10 diseases associated with DM using the topology-based measure and FunDO as the source of disease-gene associations. Results obtained by other measures and data are not shown here due to space limitations.

**Table 5:** **List of the top 10 diseases associated with DM**

Rank	Code	Disease name	Reference
1	239	Neoplasms of unspecified	PMID: 23639840
		nature	
2	155	Malignant neoplasm of liver	GWAS
		and intrahepatic bile ducts	
3	710	Diffuse diseases of connective	GWAS
		tissue	
4	714	Rheumatoid arthritis and other	GWAS
		inflammatory polyarthropathies	
5	256	Ovarian dysfunction	ICD-9, GWAS
5	278	Overweight, obesity and	ICD-9, comorbidity,
		other hyperalimentation	GWAS
7	401	Essential hypertension	Comorbidity
8	295	Schizophrenic disorders	PMID: 17474808
9	282	Hereditary hemolytic anemias	GWAS
10	289	Other diseases of blood and	PMID: 11727971
		blood-forming organs	

The top 10 diseases associated with DM were inferred using the topology-based similarity measure and FunDO as the source of disease-gene associations. Only diseases annotated in all four disease-gene association datasets are listed in the table. For a disease associated with DM according to ICD-9, comorbidity or GWAS, we added the supported evidence to the reference (the last column). The remaining disease associations were validated via mining the literature on PubMed (http://www.ncbi.nlm.nih.gov/pubmed), and for each disease only one reference (shown by PubMed ID) was listed in the table due to space limitation.

Among these 10 diseases, both ovarian dysfunction (ICD-9 code: 256) and obesity (ICD-9 code: 278) are classified under the same ICD-9 catalogue ‘Endocrine, nutritional and metabolic diseases, and immunity disorders’ with DM. In addition, both obesity and essential hypertension (ICD-9 code: 401) have highly confident comorbidity associations with DM. Note that among all disease pairs that we analysed, only 0.74% of them have a *ϕ*-correlation score higher than 0.06. Therefore, the *ϕ*-correlation scores reported in the case study (see Additional file 1: Table S4 and Additional file 1: Table S5 for details) are relatively high compared with the *ϕ*-correlation scores of all disease pairs. Moreover, 6 out of 10 associations are supported by the GWAS data, e.g., rheumatoid arthritis shares 8 genes with DM according to GWAS data. Apart from the above, associations between DM and the remaining diseases listed in the table are considered as novel associations predicted by the topology-based measure. We evaluated the top 14 novel associations via mining the literature on PubMed (see Additional file 1: Table S4 for details). We are able to confirm all of these associations, including surprising associations such as DM and ‘other cerebral degenerations’ (ICD-9 code: 331). This result highlights the power of our approaches to identify novel associations between diseases. Further exploration of potential underlying mechanisms shared by these diseases may lead to improvement in disease diagnosis, prognosis and treatment.

Another case study (Parkinson’s disease, ICD-9 code: 332) can be found in the Additional file 1.

## Conclusions

In this study, we gained novel insights into the relationship between human diseases by considering their molecular causes and underlying physical interactions. We used information derived from latest biological data, including disease-gene associations, gene functions and the topology of the human PPI network in our analysis. We applied three different measures to estimate the similarity score of diseases, and these measures were systematically evaluated against ICD-9 classification system, a statistical measure of comorbidity and GWAS data. Our results showed the correlation between associations predicted by our measures and known disease associations, and we also demonstrated the use of our measures in discovering novel disease associations and validated it via literature curation.

Novel disease associations uncovered in this study can be further used to improve our understanding of disease classification. For example, a human disease network that models the relationship of diseases can be constructed based on these similarity measures, and computational approaches, such as clustering, can be applied to detect communities in the disease network. This may provide the opportunity to redefine the current disease classification and further lead to improvements in disease diagnosis, prognosis and treatment.

## Electronic supplementary material

Additional file 1: **Supplementary information.** The Supplementary information file contains all additional figures and tables mentioned in the manuscript. (PDF 501 KB)

## Abbreviations

GWAS: Genome-Wide Association Studies
OMIM: Online Mendelian Inheritance in Man
ICD: International Classification of Diseases
PPI: Protein-Protein Interaction
GO: Gene Ontology
IEA: Inferred from Electronic Annotation
CTD: Comparative Toxicogenomics Database
FunDO: Functional Disease Ontology
HuGENet: Human Genome Epidemiology Network
MeSH: Medical Subject Heading
GeneRIF: Gene Reference Into Function
DO: Disease Ontology
SNP: Single-Nucleotide Polymorphism
ROC: Receiver Operating Characteristic
TPR: True Positive Rate
FPR: False Positive Rate
AUC: Area Under Curve
DM: Diabetes Mellitus.
