# Supplementary material for: Predicting disease associations via biological network analysis
Source: BMC Bioinformatics. 2014 Sep 17;15(1):304. doi: 10.1186/1471-2105-15-304 (PMC4174675; doi:10.1186/1471-2105-15-304)
Supplement: Supplementary file 1 — Additional file 1: Supplementary information. The Supplementary information file contains all additional figures and tables mentioned in the manuscript. (PDF 501 KB) [file 12859_2013_6584_MOESM1_ESM.pdf]

Supplementary Information

Kai Sun, Joana Gonçalves, Chris Larminie, and Nataša Pržulj

1. Supplementary Figures

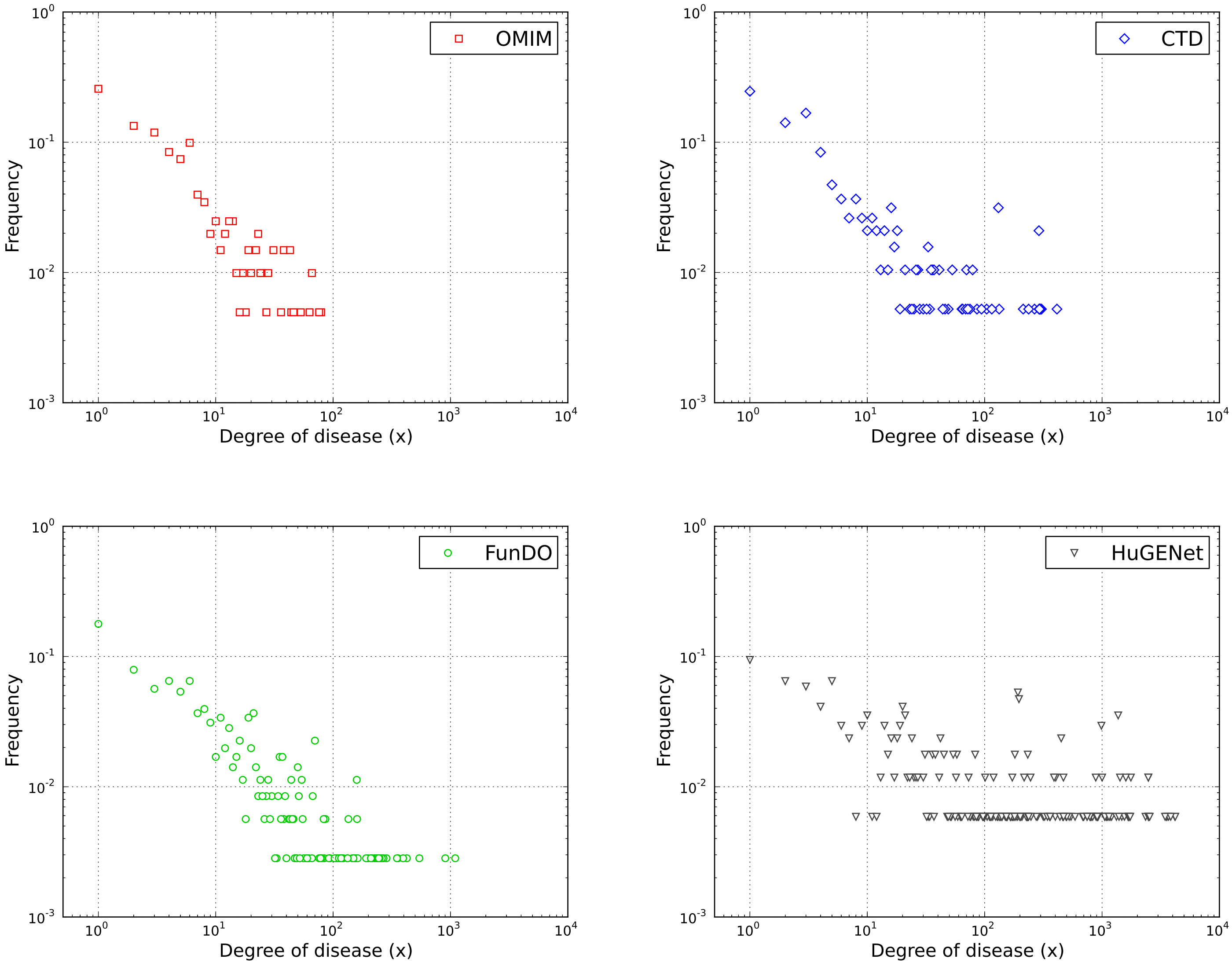

Figure S1: Degree distributions of diseases of the four disease-gene association datasets we analysed.

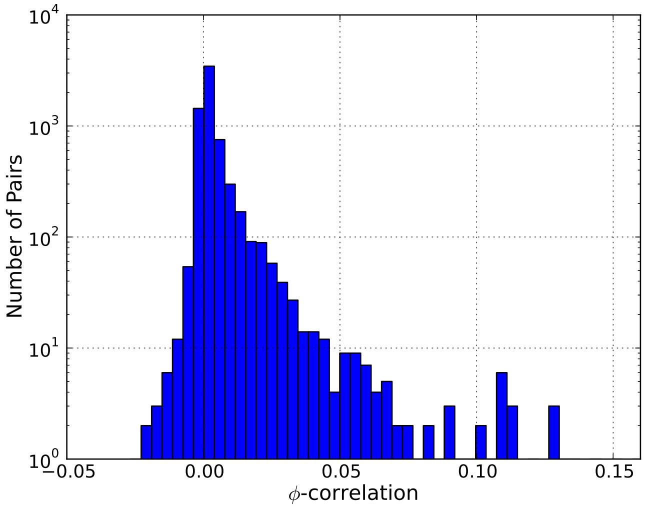

Figure S2: The distribution of ϕ-correlation scores.

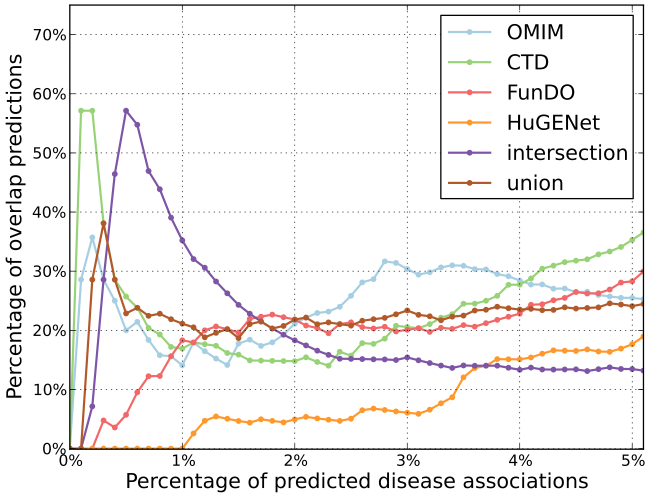

Figure S3: The overlap of predictions. The x-axis shows the percentage of predicted associations in all disease pairs we analysed, and the y-axis shows the percentage of overlap among associations predicted by the three similarity measures.

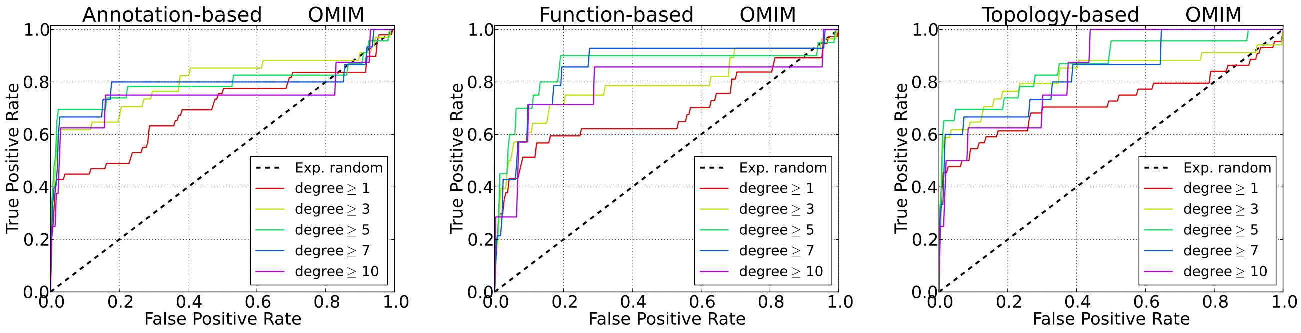

Figure S4: ROC curves obtained by evaluating the three disease similarity measures against comorbidity. OMIM (downloaded in June 2014) was used as the disease-gene association dataset. The PPI data obtained from BioGRID version 3.2.112 was used to compute the topology-based similarity scores. The ϕ-correlation threshold was set to 0.06. For each similarity measure, we evaluated diseases annotated with at least 1, 3, 5, 7, 10, 15 genes, showing by curves with different colours in each plot.

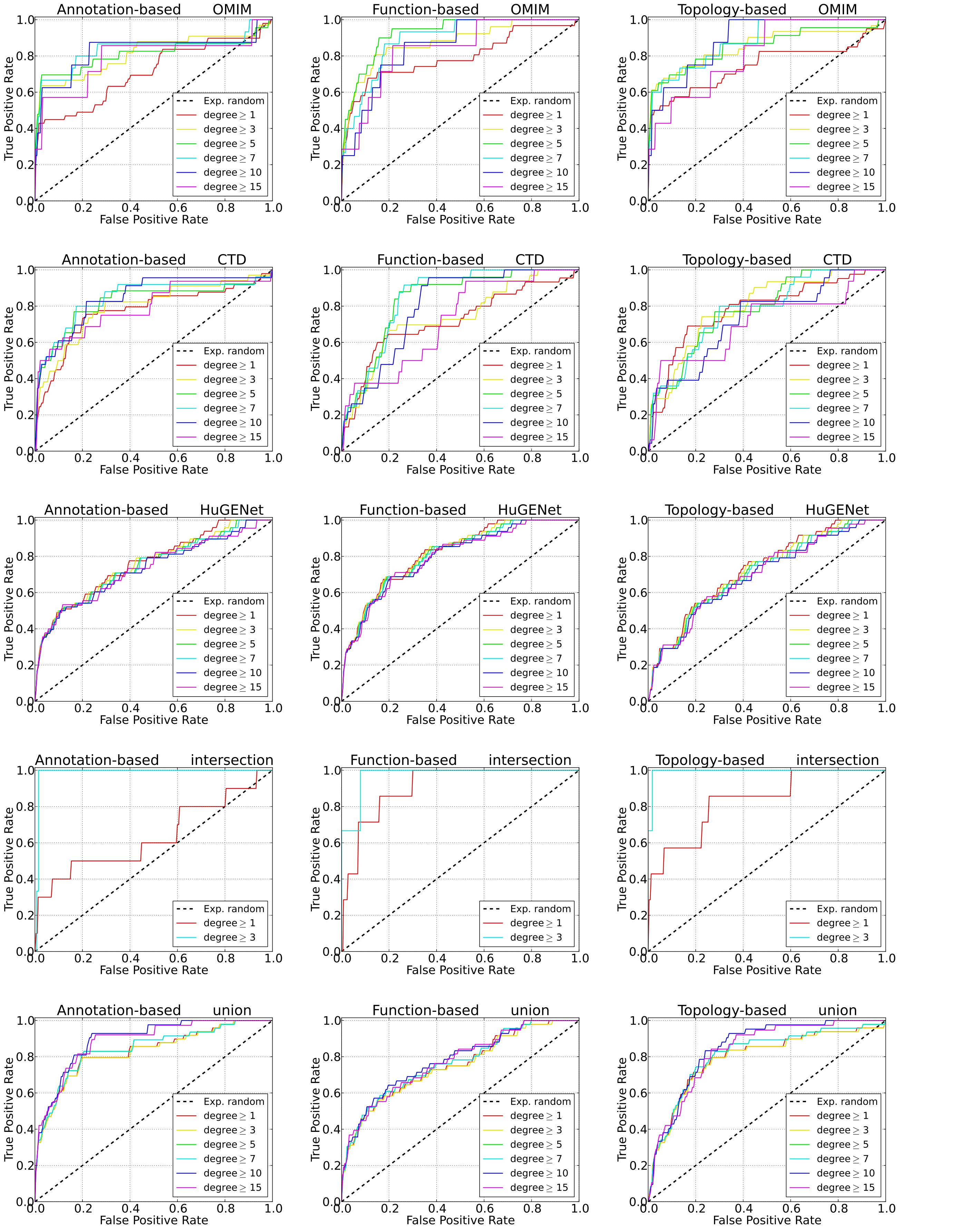

Figure S5: ROC curves obtained by evaluating the three disease similarity measures against comorbidity. The ϕ-correlation threshold was set to 0.06. For each combination of disease-gene association data and disease similarity measure, we evaluated diseases annotated with at least 1, 3, 5, 7, 10, 15 genes, showing by curves with different colours in each plot. For the intersection set, only two curves were shown in each plot since there were no comorbidity relationships between diseases annotated with more than 5 genes.

2. Supplementary Table

PPI Data	Nodes	Edges
BioGRID version 2.0.56 (released in September 2009)	6,098	18,744
BioGRID version 3.0.68 (released in September 2010)	8,433	29,971
BioGRID version 3.1.80 (released in September 2011)	9,056	37,640
BioGRID version 3.1.93 (released in October 2012)	11,261	66,253

Table S1: PPI data obtained from different versions of BioGRID database.

Data	Version 2.0.56	Version 3.0.68	Version 3.1.80	Version 3.1.93
OMIM	0.7222 ± 0.0009	0.7431 ± 0.0008	0.7262 ± 0.0007	0.8495 ± 0.0011
CTD	0.7669 ± 0.0033	0.7524 ± 0.0034	0.7569 ± 0.0041	0.7949 ± 0.0042
FunDO	0.7451 ± 0.0028	0.7401 ± 0.0022	0.7274 ± 0.0026	0.7497 ± 0.0016
HuGENet	0.6853 ± 0.0015	0.6965 ± 0.0021	0.7099 ± 0.0017	0.7153 ± 0.0015
Intersection	0.9993 ± 0.0045	0.9965 ± 0.0034	0.9962 ± 0.0039	0.9958 ± 0.0041
Union	0.7605 ± 0.0027	0.7685 ± 0.0020	0.7688 ± 0.0021	0.7939 ± 0.0022

Table S2: AUC values obtained by evaluating the topology-based similarity measure against comorbidity, using PPI data obtained from different versions of BioGRID database. The ϕ-correlation threshold was set to 0.06 and all diseases annotated with least 3 genes were evaluated. Each evaluation test was run 30 times to compute the statistics reported in the table.

	ShareSig	AllSig
OMIM	0.6657 ± 0.0369	0.8449 ± 0.0019
CTD	0.6452 ± 0.0369	0.7785 ± 0.0089
FunDO	0.7485 ± 0.0168	0.6480 ± 0.0161
HuGENet	0.7154 ± 0.0028	0.5654 ± 0.0416

Table S3: Evaluation of the topology-based similarity against comorbidity. AUC values were obtained by using $AllSig$ and $ShareSig$ as the topology-based similarity scores.

Rank	Code	Disease name	ϕ-correlation	GWAS	Score	Reference
1	239	Neoplasms of unspecified nature	0.0029	–	0.9917 (99.98%)	PMID: 23639840
2	155	Malignant neoplasm of liver and intrahepatic bile ducts	0.0047	1	0.9881 (99.87%)	GWAS
3	710	Diffuse diseases of connective tissue	-0.0019	1	0.9872 (99.76%)	GWAS
4	714	Rheumatoid arthritis and other inflammatory polyarthropathies	-0.0098	8	0.9867 (99.61%)	GWAS
5	**256**	Ovarian dysfunction	0.0001	4	0.9850 (99.56%)	ICD-9, GWAS
5	**278**	Overweight, obesity and other hyperalimentation	**0.0833**	3	0.9850 (99.56%)	ICD-9, comorbidity, GWAS
7	401	Essential hypertension	**0.1275**	0	0.9845 (99.55%)	Comorbidity
8	295	Schizophrenic disorders	0.0069	0	0.9819 (99.32%)	PMID: 17474808
9	282	Hereditary hemolytic anemias	0.0060	1	0.9818 (99.25%)	GWAS
10	289	Other diseases of blood and blood-forming organs	0.0005	–	0.9811(99.24%)	PMID: 11727971
11	642	Hypertension complicating pregnancy childbirth and the puerperium	0.0006	–	0.9795 (99.17%)	PMID: 18558027
12	365	Glaucoma	0.0150	1	0.9779 (98.91%)	GWAS
13	135	Sarcoidosis	0.0048	0	0.9778 (98.91%)	PMID: 23075651
13	414	Other forms of chronic ischemic heart disease	**0.1106**	3	0.9778 (98.91%)	Comorbidity, GWAS
15	331	Other cerebral degenerations	-0.0038	0	0.9740 (98.63%)	PMID: 20837967
15	332	Parkinson's disease	-0.0032	0	0.9740 (98.63%)	PMID: 23335160
17	**244**	Acquired hypothyroidism	0.0193	0	0.9730 (98.61%)	ICD-9
18	335	Anterior horn cell disease	0.0008	0	0.9720 (98.47%)	PMID: 22017321
19	362	Other retinal disorders	**0.0853**	0	0.9716 (98.46%)	Comorbidity
20	753	Congenital anomalies of urinary system	-0.0026	–	0.9714 (98.42%)	PMID: 22260488
21	**277**	Other and unspecified disorders of metabolism	0.0003	0	0.9708 (98.39%)	ICD-9
22	286	Coagulation defects	0.0076	–	0.9679 (98.26%)	PMID: 22460041
23	042	Human immunodeficiency virus [HIV] disease	-0.0003	0	0.9678 (98.24%)	PMID: 19419710
24	340	Multiple sclerosis	-0.0040	5	0.9649 (98.00%)	GWAS
24	579	Intestinal malabsorption	0.0015	7	0.9649 (98.00%)	GWAS
26	**272**	Disorders of lipoid metabolism	0.0342	–	0.9644 (97.79%)	ICD-9
27	577	Diseases of pancreas	0.0079	0	0.9643 (97.78%)	PMID: 22996690
28	287	Purpura and other hemorrhagic conditions	0.0132	–	0.9636 (97.60%)	PMID: 21092704
28	290	Dementias	0.0034	0	0.9636 (97.60%)	PMID: 23543134
28	581	Nephrotic syndrome	0.0544	–	0.9636 (97.60%)	PMID: 16995591

Table S4: List of the top 30 diseases associated with DM. The similarity scores (the 6th column, denoted by 'Score') were computed by using the topology-based measure and FunDO was used as the source of disease-gene associations. Only diseases annotated in all four disease-gene association datasets are listed in the table. For a disease associated with DM according to ICD-9, we highlighted its ICD-9 code (the 2nd column, denoted by 'Code') in bold and added 'ICD-9' to the reference (the last column). For a disease associated with DM according to comorbidity, we highlighted the ϕ-correlation score in bold and added 'comorbidity' to the reference. For each disease, we listed the number of genes it shared with DM according to GWAS data in the 5th column ('-' indicates no significant associations were reported in GWAS data for that disease). The remaining disease associations were validated via text mining the literature on PubMed, and for each disease only one reference (shown by PubMed ID) was listed in the table due to space limitation.

Rank	Code	Disease name	ϕ-correlation	GWAS	Score	Reference
1	239	Neoplasms of unspecified nature	0.0030	–	0.9877 (99.84%)	PMID: 22278152
2	**331**	Other cerebral degenerations	**0.0623**	0	0.9833 (99.38%)	ICD-9, Comorbidity
3	250	Diabetes mellitus	-0.0032	0	0.9740 (98.63%)	PMID: 23335160
3	256	Ovarian dysfunction	0.0003	0	0.9740 (98.63%)	PMID: 15351195
5	714	Rheumatoid arthritis and other inflammatory polyarthropathies	-0.0018	0	0.9739 (98.63%)	PMID: 18525447
5	155	Malignant neoplasm of liver and intrahepatic bile ducts	-0.0010	0	0.9739 (98.63%)	PMID: 24148818
7	290	Dementias	**0.0843**	0	0.9725 (98.52%)	Comorbidity
8	202	Other malignant neoplasms of lymphoid and histiocytic tissue	-0.0024	1	0.9689 (98.29%)	GWAS
9	**335**	Anterior horn cell disease	0.0084	0	0.9666 (98.20%)	ICD-9
9	758	Chromosomal anomalies	0.0004	–	0.9666 (98.20%)	PMID: 23162423
11	216	Benign neoplasm of skin	-0.0007	–	0.9661 (98.13%)	–
11	401	Essential hypertension	-0.0022	0	0.9661 (98.13%)	PMID: 9403584
11	642	Hypertension complicating pregnancy childbirth and the puerperium	0.0005	–	0.9661 (98.13%)	–
14	710	Diffuse diseases of connective tissue	-0.0009	1	0.9645 (97.80%)	GWAS
15	**340**	Multiple sclerosis	-0.0010	1	0.9552 (96.81%)	ICD-9, GWAS
16	300	Anxiety, dissociative and somatoform disorders	0.0216	–	0.9442 (95.73%)	PMID: 20479358
16	301	Personality disorders	0.0179	–	0.9442 (95.73%)	PMID: 22083431
16	305	Nondependent abuse of drugs	-0.0082	0	0.9442 (95.73%)	PMID: 20443774
16	307	Special symptoms or syndromes not elsewhere classified	0.0135	0	0.9442 (95.73%)	PMID: 18181204
20	277	Other and unspecified disorders of metabolism	0.0011	0	0.9424 (95.51%)	PMID: 21645034
21	**365**	Glaucoma	0.0068	0	0.9421 (95.50%)	ICD-9
22	295	Schizophrenic disorders	0.0337	1	0.9406 (95.46%)	GWAS
22	**333**	Other extrapyramidal disease and abnormal movement disorders	**0.0708**	1	0.9406 (95.46%)	ICD-9, Comorbidity, GWAS
24	**362**	Other retinal disorders	-0.0021	0	0.9356 (95.20%)	ICD-9
25	577	Diseases of pancreas	-0.0023	0	0.9194 (94.47%)	PMID: 22745701
26	278	Overweight, obesity and other hyperalimentation	-0.0094	0	0.9135 (94.21%)	PMID: 23175195
26	783	Symptoms concerning nutrition metabolism and development	0.0175	–	0.9135 (94.21%)	PMID: 17131227
28	414	Other forms of chronic ischemic heart disease	0.0013	2	0.9100 (93.95%)	GWAS
28	733	Other disorders of bone and cartilage	0.0168	0	0.9100 (93.95%)	PMID: 23000281
30	042	Human immunodeficiency virus [HIV] disease	-0.0008	0	0.9036 (93.29%)	PMID: 19748551

Table S5: List of the top 30 diseases associated with Parkinson's disease (PD).The association scores (the sixth column, denoted by 'Score') were computed by using the topology-based measure and FunDO was used as the source of disease-gene associations. Only diseases annotated in all four disease-gene association datasets are listed in the table. For a disease associated with PD according to ICD-9, we highlighted its ICD-9 codes (the second column, denoted by 'Code') in bold and put 'ICD-9' as the reference (the last column). For a disease associated with PD according to comorbidity, we highlighted the ϕ-correlation score in bold and put 'comorbidity' as the reference. For each disease, we listed the number of genes it shared with PD in the fifth column ('-' indicates that no GWAS studies have been conducted or no highly significant associations were collected in NHGRI GWAS catalog for that disease). The remaining disease associations were validated via text mining the literature on PubMed, and for each disease only one reference (shown by PubMed ID) was listed in the table due to space limitation. If a disease association haven't been uncovered in the literature, it was marked with '-' in the last column of the table.
